# Effects of Kisspeptin Administration in Women With Hypoactive Sexual Desire Disorder: A Randomized Clinical Trial

**DOI:** 10.1001/jamanetworkopen.2022.36131

**Published:** 2022-10-26

**Authors:** Layla Thurston, Tia Hunjan, Natalie Ertl, Matthew B. Wall, Edouard G. Mills, Sofiya Suladze, Bjial Patel, Emma C. Alexander, Beatrice Muzi, Paul A. Bassett, Eugenii A. Rabiner, Paul Bech, David Goldmeier, Ali Abbara, Alexander N. Comninos, Waljit S. Dhillo

**Affiliations:** 1 Section of Endocrinology and Investigative Medicine, Imperial College London, London, United Kingdom; 2 Invicro, a Konica Minolta company, London, United Kingdom; 3 Statsconsultancy Ltd., Amersham, United Kingdom; 4 Department of Sexual Medicine, Imperial College Healthcare NHS Trust, London, United Kingdom; 5 Department of Endocrinology, Imperial College Healthcare NHS Trust, London, United Kingdom

## Abstract

**Importance:**

Despite being the most common female sexual health complaint worldwide, current treatment options for hypoactive sexual desire disorder (HSDD) are limited in their safety and effectiveness. The hormone kisspeptin is a key endogenous activator of the reproductive hormonal axis with additional emerging roles in sexual and emotional behavior; however, its effects in women with HSDD are unknown.

**Objective:**

To test the hypothesis that kisspeptin enhances sexual and attraction brain processing in women with HSDD.

**Design, Setting, and Participants:**

This randomized clinical trial was double-masked and placebo controlled with a 2-way crossover. The trial was conducted in a university research setting in the UK from October 2020 to April 2021. Eligible participants were premenopausal women with HSDD. Functional neuroimaging, psychometric, and hormonal analyses were employed to investigate the effects of kisspeptin administration on brain processing, in response to erotic stimuli (erotic videos) and facial attraction (face images of varying attractiveness). Data were analyzed from May to December 2021.

**Interventions:**

A 75-minute intravenous infusion of kisspeptin-54 (1 nmol/kg/h) vs equivalent-rate placebo infusion.

**Main Outcomes and Measures:**

Blood oxygen level–dependent responses across the whole brain and regions of interest during kisspeptin vs placebo administration in response to erotic and facial attraction stimuli.

**Results:**

Of the 40 participants who were randomized, 32 women completed both kisspeptin and placebo visits, with a mean (SE) age of 29.2 (1.2) years. Kisspeptin administration resulted in modulations in sexual and facial attraction brain processing (deactivation of the left inferior frontal gyrus: *Z* max, 3.76; *P* = .01; activation of the right postcentral and supramarginal gyrus: *Z* max, 3.73; *P* < .001; deactivation of the right temporoparietal junction: *Z* max 4.08; *P* = .02). Furthermore, positive correlations were observed between kisspeptin-enhanced hippocampal activity in response to erotic videos, and baseline distress relating to sexual function (*r* = 0.469; *P* = .007). Kisspeptin’s enhancement of posterior cingulate cortex activity in response to attractive male faces also correlated with reduced sexual aversion, providing additional functional significance (*r* = 0.476, *P* = .005). Kisspeptin was well-tolerated with no reported adverse effects.

**Conclusions and Relevance:**

These findings lay the foundations for clinical applications for kisspeptin in women with HSDD.

**Trial Registration:**

ISRCTN trial registry identifier: ISRCTN17271094

## Key Points

**Question:**

Does kisspeptin administration improve sexual and attraction brain processing in women with hypoactive sexual desire disorder?

**Findings:**

In this randomized clinical trial including 32 premenopausal women with hypoactive sexual desire disorder, kisspeptin administration was found to modulate sexual and attraction brain processing in functional neuroimaging, psychometric, and hormonal analyses. Furthermore, kisspeptin’s modulation of brain processing correlated with psychometric measures of sexual aversion and associated distress.

**Meaning:**

These findings lay the foundations for the clinical applications of kisspeptin in women with hypoactive sexual desire disorder.

## Teaser

This randomized clinical trial of 32 premenopausal women with hypoactive sexual desire disorder examines the effectiveness of a kisspeptin infusion in enhancing sexual and attraction brain processing.

## Introduction

Sexual behavior is important for overall health and well-being. Sexual desire is a key component of the human sexual response.^1^ Absence or deficiency of sexual desire can cause marked distress or interpersonal difficulty, leading to what has been defined in the *Diagnostic and Statistical Manual of Mental Disorders* (Fourth Edition) as hypoactive sexual desire disorder (HSDD).^2^ HSDD is very common, affecting 10% of women,^3^ yet despite the psychological and economic burden there are surprisingly limited treatment options available.^4^ Indeed, widespread use of the 2 licensed medications in the US for premenopausal women with HSDD, flibanserin (5-HT_1A_ agonist/5-HT_2A_ antagonist) and bremelanotide (melanocortin-4 receptor agonist), is limited by their adverse-effect burden, including dizziness and nausea, as well as their limited effectiveness.^5,6^ Several other neurotransmitters have been investigated as potential therapeutic targets for HSDD, namely dopamine,^7^ noradrenaline,^8^ and oxytocin.^9^ However, clinical trials have thus far failed to provide new licensed therapies based on these compounds. There is therefore a significant clinical unmet need to identify novel, safe, and effective therapeutic targets to address the considerable burden of HSDD.

One of the barriers to treatment development is that the etiology of HSDD is not fully understood. HSDD is thought to be multifaceted, with neuroendocrine, psychological, and behavioral processes playing roles. The introduction of functional magnetic resonance imaging (fMRI) in the study of HSDD has made an important contribution to our understanding of female sexual function. fMRI studies examining brain activity in women with HSDD have identified brain regions of complementary hyperactivation and hypoactivation in response to erotic stimuli. Analysis of the functional roles of these regions has informed the so-called “top-down” theory of HSDD, whereby during sexual stimuli there is increased activity of higher cortical and cognitive regions, which inhibit the lower limbic and emotional regions, thus interfering with sexual desire.^10^

A potential novel treatment target is the hormone kisspeptin (encoded by the KISS1 gene), which is a key endogenous hypothalamic activator of the reproductive hormone axis. However, recent data suggest additional key extrahypothalamic roles in sexual and emotional behavior.^11,12,13,14,15,16^ Kisspeptin and its receptor (encoded by the KISS1R gene) are widely expressed in limbic behavioral brain regions.^17,18,19,20,21^ In keeping with this, preclinical data suggest that kisspeptin acts on brain pathways controlling reproductive behaviors in male^11,22^ and female^12^ rodents. Furthermore, previous work in healthy men has demonstrated that kisspeptin enhances limbic brain activity in response to sexual stimuli.^13^

Together, these previous data led us to hypothesize that kisspeptin has translational potential in women with HSDD. To test our hypothesis, we used psychometric, functional neuroimaging and hormonal assessments to investigate the effects of kisspeptin administration on sexual and attraction brain processing in 32 premenopausal women with HSDD.

## Methods

### Trial Design

This randomized, double-masked, 2-way crossover, placebo-controlled clinical trial was preregistered on ISRCTN trial registry (identifier ISRCTN17271094) (Supplement 1; eMethods in Supplement 2). This report follows the Consolidated Standards of Reporting Trials (CONSORT) reporting guideline for randomized studies. Ethical approval for this trial was provided by the Riverside Research Ethics Committee. All participants provided written informed consent.

**Figure 1.**
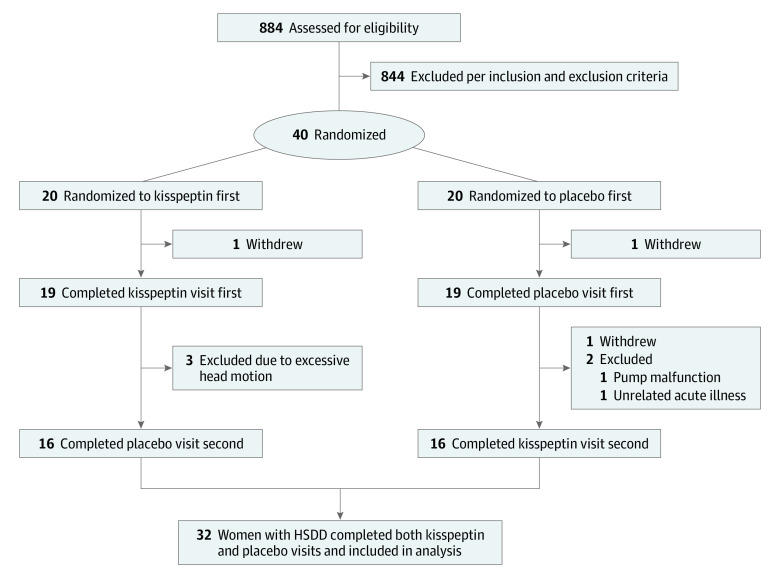
Trial Participation Diagram HSDD indicates hypoactive sexual desire disorder.

**Figure 2.**
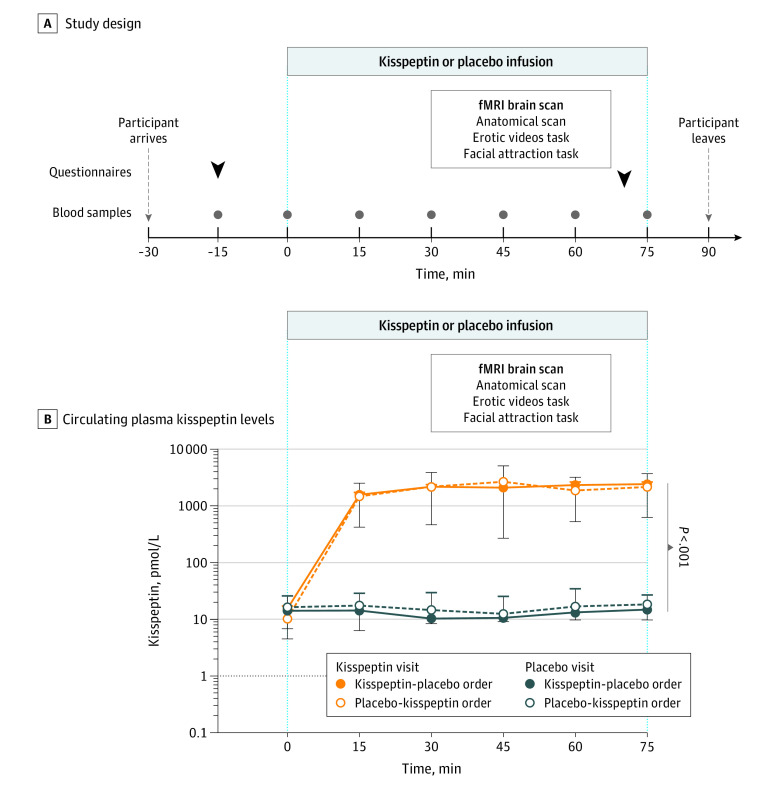
Experimental Protocol and Effects of Kisspeptin Administration on Circulating Kisspeptin Levels Participants attended 2 double-masked study visits at least 1 month apart (to allow full washout) in days 1 to 7 of the menstrual cycle: 1 for intravenous infusion of kisspeptin (1 nmol/kg/h) and 1 for intravenous infusion of equivalent volume of placebo for 75 minutes, in random order. In panel A, arrows indicate time points at which participants completed the following baseline and post-scan questionnaires: the Sexual Arousal and Desire Inventory, the State-Trait Anxiety Inventory Form Y-1 and the d2 Test of Attention. Participants underwent a functional MRI (fMRI) brain scan and the following types of scan and tasks were administered: anatomical (to evaluate any structural abnormality and for subsequent anatomical location), erotic videos task (watching 10 20-second short erotic videos with 10 20-second exercise control videos) and a facial attraction task (male and female faces of medium and high attractiveness). Dots indicate time points at which blood samples were taken for reproductive hormone levels. In panel B, kisspeptin administration resulted in increased circulating plasma kisspeptin levels.

Heterosexual premenopausal women concerned by low sexual desire were invited to take part via advertisements (Figure 1). Interested participants were initially telephone-screened and subsequently underwent a detailed medical screening visit which was supervised by an experienced consultant in sexual medicine (D.G.). Participants attended twice and received the alternative treatment (kisspeptin or placebo) on their second visit at least 1 month later (Figure 2A). Sixteen participants received kisspeptin and 16 received placebo on their first visit. Each participant acted as their own control to maximize study power. The studies were conducted on days 1 through 7 of the menstrual cycle (ie, the follicular phase) as brain activation on viewing erotic stimuli can vary in different phases of the cycle.^23^

Participants, study doctors, research nurses, radiographers and data analysts were masked as to the infusion identity, the order of which was randomized using Randomizer.org in a balanced manner. One author (E.M.) prepared the kisspeptin and placebo infusion (and thus was unmasked); however, this author did not have any contact with the participants or involvement in data analysis. Intravenous infusions of kisspeptin or placebo were identical in appearance, volume, and rate, and were infused in identical syringe pumps.

### Outcome Measures

Changes in task-related (erotic stimuli and facial attraction) blood oxygen level-dependent (BOLD) activity on fMRI when participants are administered kisspeptin compared with placebo. Secondary outcomes included correlation analyses between BOLD activity and psychometric and behavioral parameters; changes in psychometric and behavioral measures of sexual desire and arousal, state anxiety, and nonsexual attention; and changes in hormone levels (plasma kisspeptin and serum luteinizing hormone [LH], follicle stimulating hormone [FSH], estradiol, progesterone, and testosterone) during kisspeptin compared with placebo administration.

### Statistical Analysis

Statistical analyses, including the power calculation, were designed in collaboration with an independent statistician (P.A.B.) and were conducted using Prism version 9.3 (GraphPad Software). Sample size was calculated using data from a previous fMRI study examining the effects of kisspeptin on fMRI sexual brain activity in healthy men,^13^ as no such similar study has been performed in women. This study showed that kisspeptin enhances task-related BOLD activity on fMRI by a mean (SD) 0.74% (0.38%) compared with placebo (mean [SD], 0.48% [0.51%]). Using these data, and assuming a similar effect in women with HSDD, with 5% significance level and 80% power, and assuming a correlation coefficient of 0.4 between kisspeptin and placebo, a power calculation was performed resulting in a sample size of 31. To allow for natural variation in responses, drop-out, and exclusion of approximately 20%, 40 participants were recruited to the study. In addition, this sample size is in keeping with empirically derived estimates to allow sufficient power to detect moderate-sized effects in fMRI studies,^24^ as well as noninterventional fMRI studies in women with HSDD^25^ and our previous work examining the hormonal effects of kisspeptin vs placebo on brain activity in healthy volunteers.^13,14,15,26,27^ Wilcoxon matched-pairs signed rank tests were used to determine differences between psychometric scores (SADI [Sexual Arousal and Desire Inventory], STAI [State-Trait Anxiety Inventory] Y-1, and d2) between kisspeptin and placebo visits. Reproductive hormones were analyzed using a repeated measures 2-way ANOVA. A single-group paired difference (paired *t* test) was constructed to test the differences between kisspeptin and placebo in the erotic video and facial attraction task. Pearson correlation was used to assess correlations between region of interest brain activity during the tasks and psychometric and behavioral parameters. To adjust for the number of analyses, a reduced α threshold from standard *P* < .05 to *P* < .01 identified statistical significance in the correlation analyses, in line with previous work.^13,14^

## Results

Of the 40 participants who were randomized, 32 women completed both kisspeptin and placebo visits. Mean (SE) age was 29.2 (1.2) years; 3 participants (9%) were Asian, 2 (6%) Black, 2 (6%) Hispanic, and 25 (78%) White (Table).

**Table.** Baseline Participant Characteristics

Characteristic	Mean (SE)			
	Kisspeptin-placebo order		Placebo-kisspeptin order	
**Demographics**				
Age, y	30.4 (1.4)		27.9 (1.9)	
BMI	23.5 (0.8)		22.8 (0.6)	
Race or ethnicity, No. (% of group)				
Asian	2 (13)		1 (6)	
Black	2 (13)		0	
Hispanic	0		2 (13)	
White	12 (75)		13 (81)	
**Sexual history**				
Age of partner, y	31.6 (2.0)		29.8 (2.2)	
Duration of relationship, mo	72.1 (10.2)		51.1 (10.7)	
Intercourse per month	2.3 (0.4)		2.8 (0.4)	
Duration of distressing low sexual desire (months)	45.6 (8.9)		37.6 (7.8)	
FSFI^a^				
Total (range, 2.0-36.0)	16.8 (1.7)		19.4 (1.2)	
Desire domain (range, 1.2-6.0)	1.6 (0.1)		2.0 (0.2)	
FSDS-DAO^b^				
Total (range, 0-60)	41.1 (2.1)		38.3 (2.1)	
Item 13 (range, 0-4)	3.5 (0.2)		3.6 (0.1)	
Depression and anxiety questionnaires				
PHQ-9 (range, 0-27)^c^	2.8 (0.7)		1.8 (0.4)	
GAD-7 (range, 0-21)^d^	2.9 (0.6)		2.3 (0.6)	
**Reproductive hormones**	**Kisspeptin visit**	**Placebo visit**	**Kisspeptin visit**	**Placebo visit**
Kisspeptin, pmol/L	15.1 (2.1)	14.2 (2.3)	10.3 (1.4)	16.4 (2.4)
Luteinizing hormone, IU/L	4.4 (0.8)	4.6 (0.9)	4.5 (0.8)	4.0 (0.5)
Follicle stimulating hormone, IU/L	5.1 (0.6)	4.5 (0.4)	4.9 (0.5)	4.7 (0.4)
Estradiol, pmol/L	150 (36.8)	172 (37.8)	175.1 (29.8)	201.6 (46.1)
Progesterone, nmol/L	1.9 (0.6)	3.3 (1.2)	3.1 (1.3)	1.4 (0.3)
Testosterone, nmol/L	1.0 (0.1)	1.0 (0.1)	1.0 (0.1)	1.0 (0.1)

Abbreviations: BMI, body mass index (calculated as weight in kilograms divided by height in meters squared); FSFI, Female Sexual Function Index; GAD-7, Generalized Anxiety Disorder Assessment; PHQ-9, Patient Health Questionnaire.

^a^ Total FSFI scores of 26.0 or lower indicate sexual dysfunction; scores on the desire domain of 5.0 or lower indicate low desire.

^b^ High total FSDS-DAO scores indicate high distress; high scores on item 13 denote participants experiencing higher levels of bother by low sexual desire.

^c^ PHQ-9 scores of 5 or higher indicate depression.

^d^ GAD-7 scores of 5 or higher indicate anxiety.

### Primary Outcome

**Figure 3.**
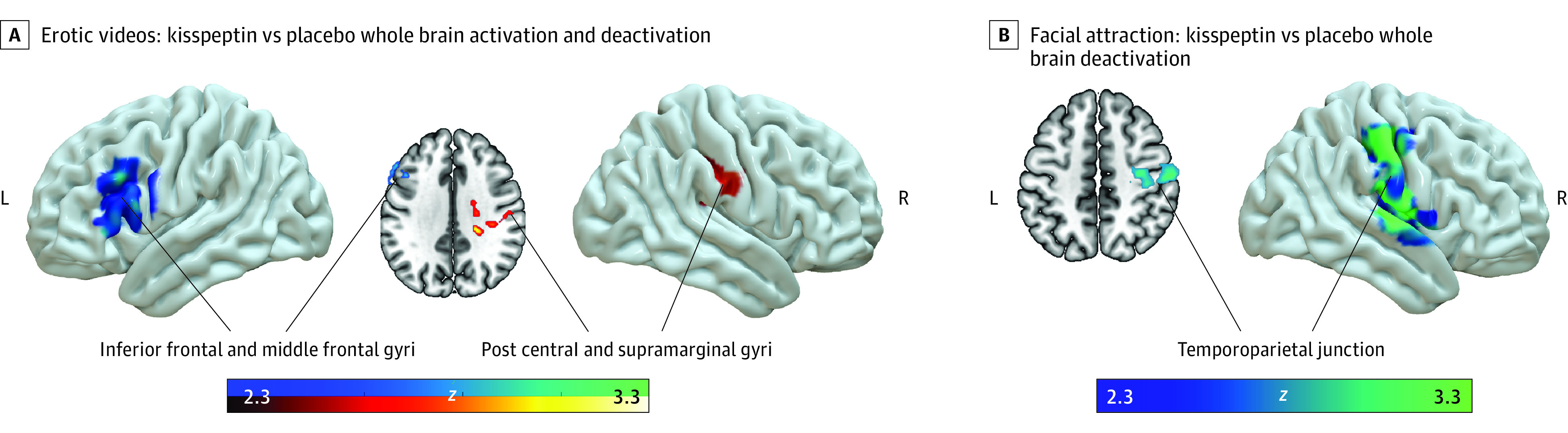
Kisspeptin vs Placebo Whole Brain Analysis During Erotic Videos and Facial Attraction Tasks Red and yellow regions indicate group activation and blue and green regions indicate group deactivation. In panel A, kisspeptin deactivated the inferior frontal and middle frontal gyri and activated the postcentral and supramarginal gyri in response to erotic compared with control exercise videos on whole brain analysis. See eFigure 1B in Supplement 2 for additional brain slices. In panel B, Kisspeptin deactivated the temporoparietal junction in response to male compared to female on whole brain analysis (blue/green regions indicate group deactivation). See eFigure 1D in Supplement 2 for additional brain slices.

The overall erotic video task effects (averaging across kisspeptin and placebo conditions) were consistent with those seen in previous studies using similar tasks, which served to validate our stimuli, tasks, and analysis procedures (*Z* max = 3.76; *P* = .01) (eFigure 1A in Supplement 2).^28^ On viewing erotic videos, kisspeptin administration deactivated the left inferior frontal gyrus extending partly to the middle frontal gyrus compared with placebo (Figure 3A; eFigure 1B in Supplement 2). Conversely, increased activation was noted in the right postcentral and supramarginal gyrus (*Z* max = 3.73; *P* < .001). Analysis using visit order as a demeaned covariate did not significantly alter the result (eFigure 2 in Supplement 2). The overall task effects in the facial attraction task (averaging across kisspeptin and placebo conditions) were consistent with those seen in a previous study using similar tasks, demonstrating effective task design (eFigure 1C in Supplement 2).^15^ Kisspeptin administration deactivated a region in the right temporoparietal junction centered on the secondary somatosensory cortex when participants viewed male compared with female faces (*Z* max = 4.08; *P* = .02) (Figure 3B; eFigure 1D in Supplement 2). A list of coordinates can be found in the eTable of Supplement 2.

### Secondary Outcomes

**Figure 4.**
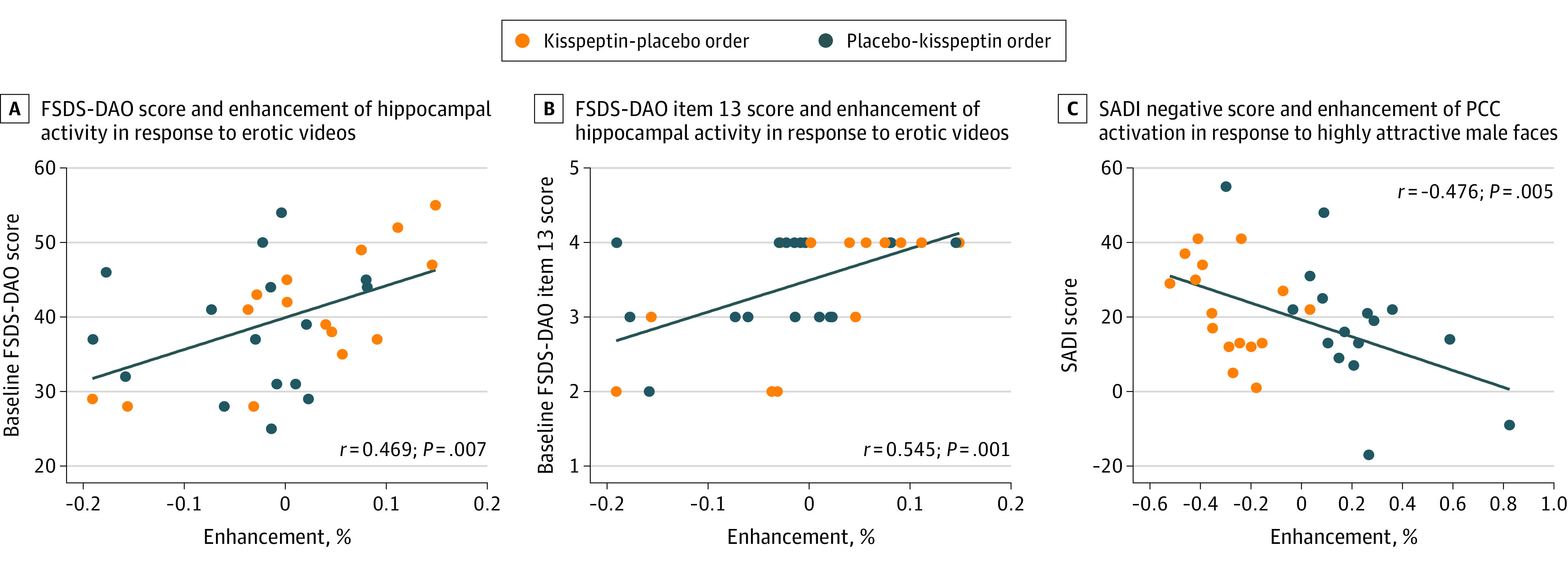
Correlation Analyses of Regions of Interest Activity With Measures of Sexual Aversion and Distress and Aversion

Participants who were more distressed by their overall sexual function (as measured on the Female Sexual Distress Scale [FSDS-DAO]^29^) showed greater kisspeptin-enhanced brain activity in the hippocampus (a key structure implicated in female sexual desire^10,30,31^) on viewing short erotic videos (*r* = 0.469; *P* = .007) (Figure 4A). Furthermore, participants who were more bothered specifically by their low sexual desire also showed increased hippocampal activity (FSDS-DAO item-13) (*r* = 0.545, *P* = .001) (Figure 4B). Correlation analysis demonstrated the more kisspeptin activated the posterior cingulate cortex in response to highly attractive male faces, the less sexual aversion reported by participants (*r* = −0.476, *P* = .005) (Figure 4C). SADI scores did not change at a domain level (eFigure 3A-D in Supplement 2). Exploratory analysis revealed a mean increase in SADI-Sexy score (worded, “How sexy do your feel right now?”) of 0.5 (95% CI, 0.05-0.95; *P* = .04) with kisspeptin administration compared with placebo (eFigure 3E in Supplement 2). Kisspeptin administration had no effect on state anxiety, assessed using the STAI Y-1 and no effect on nonsexual attention assessed using the d2 test, thereby excluding these as possible confounders for our observed results (eFigures 4A and 4B in Supplement 2). Kisspeptin administration led to a mean increase in circulating kisspeptin levels of 1305 pmol/L (95% CI, 1050 to 1560 pmol/L; *F*_1,62_ = 104.7; *P* < .001), reaching steady state levels for the duration of the study (Figure 2B). Kisspeptin administration resulted in a mean increase in LH of 2.14 IU/L (95% CI, 0.41 to 3.90 IU/L; *F*_1,62_ = 6.08; *P* = .02) and FSH of 0.28IU/L (95% CI, 0.00 to 0.55 IU/L; *F*_1,62_ = 104.7; *P* = .049), as expected (eFigures 5A and 5B in Supplement 2). There was no effect of kisspeptin administration on downstream estradiol, progesterone or testosterone, thereby excluding these possible hormonal confounders (eFigures 5C through 5E in Supplement 2). Kisspeptin was well-tolerated with no adverse effects in keeping with our previous work.^13,14,15,26^

## Discussion

HSDD causes marked psychological distress, resulting in a significant social and economic burden.^4^ Given this, and the limited effectiveness and adverse effect burden of the 2 currently available treatments, there is a significant unmet need to discover new therapeutic targets for HSDD. Our data demonstrate that in premenopausal women with HSDD, kisspeptin administration restores sexual and attraction brain processing without adverse effects.

During the erotic videos, kisspeptin administration led to deactivation of the left inferior and middle frontal gyri. The inferior frontal gyrus has a range of established functions, including processing language, working memory, and empathy,^32^ and is also implicated in inner speech, also known as internal monologue.^33,34^ Both the left inferior frontal gyrus and middle frontal gyrus are involved in inhibitory control.^35,36^ In keeping with this, an fMRI study assessing brain activation in men while they voluntarily inhibited their sexual arousal on viewing erotic videos demonstrated activation of the left inferior frontal gyrus.^37^ Moreover, a transient emotional experience of guilt also activates the left inferior frontal gyrus,^38^ which is of particular relevance given that guilt can be a feature of distress evoked by low desire in HSDD.^29^ Furthermore, it is established that women with HSDD have increased activity in the inferior frontal gyrus on viewing erotic stimuli,^31,39^ which can serve to reduce impulses and natural responses in a sexual context, thereby reducing sexual desire. The top-down theory of HSDD describes the process of excessive monitoring and self-evaluation, which can interfere with normal sexual brain processing.^10^ Taken together, and in line with this top-down theory, deactivation of the left inferior frontal and middle frontal gyri by kisspeptin can therefore reduce distracting, negative internal monologue and feelings of guilt, and permit lower-level responses to be expressed. Another finding during the erotic videos was activation of a region extending into the supramarginal and postcentral gyrus; both these latter regions have been shown to be activated in the context of sexual arousal.^25,40,41^

In the facial attraction task, kisspeptin administration led to deactivation of a distinct region in the right parietal operculum extending back into the temporoparietal junction on viewing male faces. The temporoparietal junction is linked to social-cognitive processing,^42,43^ with decreased activity in the right temporoparietal junction associated with reduced negative updating of a person’s impressions of others.^44^ Within this region lies the secondary somatosensory cortex, a region involved in high-level information processing, self-consciousness, and whole-body representation.^45^ Similar clusters around the temporoparietal junction have also been identified in interoceptive processes and functions related to body ownership.^46^ Importantly, a meta-analysis of neuroimaging studies in women with HSDD also identified small clusters in these regions as being hyperactive.^10^ In line with these studies, kisspeptin’s deactivation of this region in the current study may reduce negative perception of others and reduce self-consciousness.

Furthermore, we observed significant correlations between kisspeptin-enhanced brain activity in response to erotic stimuli and baseline distress relating to sexual function. Greater kisspeptin enhancement was observed in the hippocampus, which is a key region of the female sexual desire brain network^10,30,31^ and known to contain *KISS1* and *KISS1R*.^17,18^ More specifically, kisspeptin-enhanced hippocampal activity was greater in women who were more bothered by their low sexual desire at baseline, ie, the defining feature of HSDD.^2^ These findings suggest that kisspeptin’s enhancement of the hippocampus forms a functional mechanism for increasing sexual desire on viewing erotic stimuli in women with greater distress relating to sexual function. During the facial attraction task we observed activation of the posterior cingulate cortex (exerted by kisspeptin in response to highly attractive male faces) was correlated with reduced sexual aversion. The posterior cingulate cortex is implicated in romantic love^47^ and cognitive processes, including autobiographical memory and reward.^48,49^ Therefore, consistent with these roles, kisspeptin can serve to increase feelings of romantic love and reward processing in the posterior cingulate cortex, thereby reducing sexual aversion in women with HSDD, as observed in our study, and providing mechanistic insight.

Our results demonstrate the effects of kisspeptin administration in brain regions known to contain *KISS1R*, including the inferior and middle frontal gyri, cingulate, and hippocampus,^17,21^ which suggest a possible direct receptor-mediated action of kisspeptin in these brain regions following passage across the blood-brain barrier.^13^ Consistent with this, preclinical animal work suggests that kisspeptin-induced sexual behaviors (rodent lumbar lordosis^12^ and erections^11^) can occur independently of downstream gonadotropin-releasing hormone (GnRH) and other downstream hormones. However, we also observed effects of kisspeptin in brain regions where *KISS1R* has not yet been identified (postcentral and supramarginal gyri, and temporoparietal junction). Therefore, it is also important to consider alternate pathways that could be responsible for kisspeptin’s effects on brain activity and behavior. Although kisspeptin can also signal directly onto GnRH neurons, further studies suggest that kisspeptin also interacts with several other neuropeptides including serotonin,^50^ dopamine,^51^ gamma-aminobutyric acid,^27^ glutamate^52^ and nitric oxide.^53^ Thus, kisspeptin’s effects in this study may comprise direct effects on its cognate receptor as well as the orchestration of interactions in other neuropeptide systems.

Kisspeptin administration did not alter overall SADI domain scores. Post hoc exploratory analysis showed that kisspeptin administration increases self-reported ratings of feeling sexy compared with placebo; however, as this was an exploratory finding, confirmation is required in dedicated studies. Importantly, women with a more positive body image are more likely to desire sexual activity and gain satisfaction from sexual experiences.^54^ Furthermore, body image is a determinant factor of HSDD.^55^ Therefore, by increasing a woman’s feeling of sexiness, kisspeptin administration can drive increased sexual desire and arousal in women with HSDD. The behavioral effects of kisspeptin we report may be explained mechanistically by the observed changes in brain activation.

Kisspeptin administration led to a small increases in circulating LH and FSH levels, confirming a biologically active dose. However, it is unlikely that the behavioral and brain effects observed are due to these LH and FSH changes given the small size of the increment; moreover, LH and FSH are not known to have significant roles in sexual behavior in humans.

The underlying causes of HSDD are many, and may conceivably arise from a multitude of organic, psychological, or social (ie, relationship) factors and can be both causes or effects of HSDD. In this study we looked at primary HSDD and excluded other secondary causes such as hypogonadism, relationship factors, comorbidities, or medication-related issues. It is important to note that our study population may be somewhat heterogeneous in terms of the ultimate etiology of their HSDD; however, current theories of HSDD^56^ suggest that they are similar in the proximate causes; overactivity in brain circuits related to self-monitoring and underactivity in brain circuits related to sexual processing, and (as we have demonstrated) this mechanism may be amenable to pharmacological interventions. A useful analogy might be clinical depression, which can be caused by a multitude of interacting factors (genetic, developmental, social, psychological) but is also responsive to standard pharmacotherapies that target particular neurotransmitters and brain circuits.

### Strengths and Limitations

The strengths of this study include that it is fully powered, and that menstrual cycle and hormonal contraception were controlled for, minimizing any potential confounding effect due to fluctuations in reproductive hormone levels. Furthermore, all study visits were performed in the follicular phase of the menstrual cycle, where kisspeptin administration is known to have less of an effect on downstream sex steroids and would have confounded our findings.^57^ Indeed, our data showed that estradiol levels did not change during the study following kisspeptin administration removing this possible confounder. In addition, we assessed state anxiety and nonsexual attention, and observed no difference between kisspeptin and placebo, thus eliminating further potential confounders. The group task effects clearly demonstrate that the designed protocol robustly activated relevant sexual brain regions, confirming validity of our task design and analysis procedures. In addition, we included exercise video segments as an appropriate control for the effects of visual stimulation in the erotic videos task. Finally, the participants interacted with the same female study doctor throughout (L.T.), reducing the known risk of bias from interacting with different gender investigators.^58^

This study also had several limitations. While responses to erotic stimuli can be considered subjective and therefore somewhat variable, participants acted as their own control, which thereby minimized interparticipant variability. Moreover, an independent focus group was used to rate and select the videos that were concordant with heterosexual women’s preferred erotica.^59^ Although the results of this study are applicable to premenopausal women with HSDD, it would be important in the future to extend the study population to perimenopausal and postmenopausal women and men with HSDD. A 75-minute infusion period was employed to allow steady state kisspeptin levels to be achieved followed by sufficient time for the fMRI tasks and psychometric or behavioral assessments. It is therefore possible that earlier changes may have been missed in the initial 30 minutes of infusion before fMRI and assessments began. Furthermore, although participants were assessed during a clinical study visit involving infusions, questionnaires, and MRI scans, we may have missed more subtle effects of kisspeptin that may arise in an environment where they may have been more at ease (eg, at home).

This is the first study to examine the effects of kisspeptin on sexual behavior and brain processing in women with HSDD and therefore translates preclinical evidence into clinical findings. First, kisspeptin administration deactivated the left inferior and middle frontal gyri in response to erotic stimuli (serving to reduce the negative internal monologue and response inhibition). Second, kisspeptin deactivated the right temporoparietal junction which can reduce a woman’s focus on herself, her body image, and related negative thoughts. Third, kisspeptin-enhanced hippocampal activity was greater in women with greater overall sexual distress, but also specifically in women who were more bothered by their low desire. Finally, kisspeptin’s activation of the posterior cingulate cortex can serve to increase feelings of romantic love and reward processing, thereby reducing sexual aversion as observed.

Existing treatment options for women with HSDD are limited in their efficacy. In the current proof-of-concept study, we were investigating fMRI brain changes as a primary outcome, which has not been performed at the time of this study with the other licensed medications for HSDD and so the effects cannot be compare directly. However, the observed brain modulations by kisspeptin and correlations with psychometric results are promising from a clinical perspective, and larger clinical trials are now warranted to elucidate the therapeutic potential of kisspeptin in HSDD and related psychosexual conditions. Indeed, kisspeptin-based therapies are currently under clinical development for a range of other common reproductive disorders and in in vitro fertilization protocols.^60,61^ Recently, a kisspeptin receptor agonist in the form of a subcutaneous injection has shown promising results, with a greater duration of action on downstream hormone release than endogenous kisspeptin-54, and with no notable adverse effects.^61^ Indeed, ours and others’ clinical studies are yet to identify any notable adverse effects attributable to kisspeptin. Therefore, combining this escalation in safe kisspeptin-based therapeutics and our data demonstrating beneficial actions in women with HSDD identifies a realistic therapeutic avenue for the condition, where current treatments have limited efficacy and carry adverse effects.

## Conclusions

In summary, these data suggest that kisspeptin administration deactivates regions that are hyperactivated in women with HSDD, as well as activating additional key sexual brain regions, thereby enhancing sexual brain processing. Furthermore, kisspeptin enhances limbic brain activity that correlates with reduced sexual aversion and alters the processing of male facial attractiveness. Collectively, these findings provide key behavioral and functional relevance for kisspeptin’s enhancement of brain activity on viewing erotic stimuli and male faces and, importantly, lays the foundations for clinical applications for kisspeptin in patients with psychosexual disorders.

## Notes

Supplement 1.
Trial Protocol

Supplement 2.
eMethods.
**eTable. **Clusters With Enhanced Activation or Deactivation by Kisspeptin on Whole Brain Analysis **eFigure 1.** Kisspeptin vs Placebo Whole Brain Analysis During Erotic Videos and Facial Attraction Tasks **eFigure 2.** Erotic Videos: Kisspeptin vs Placebo Whole Brain Activation and Deactivation, Controlled for Visit Order **eFigure 3.** Effects of Kisspeptin Administration on the Sexual Arousal and Desire Inventory **eFigure 4.** Effects of Kisspeptin Administration on State Anxiety and Attention **eFigure 5.** Effects of Kisspeptin on Downstream Circulating Reproductive Hormones

Supplement 3.
Data Sharing Statement
