# Supplementary material for: Effects of Kisspeptin Administration in Women With Hypoactive Sexual Desire Disorder: A Randomized Clinical Trial
Source: JAMA Netw Open. 2022 Oct 26;5(10):e2236131. doi: 10.1001/jamanetworkopen.2022.36131 (PMC9606846; doi:10.1001/jamanetworkopen.2022.36131)
Supplement: Supplement 2. — eMethods. eTable. Clusters With Enhanced Activation or Deactivation by Kisspeptin on Whole Brain Analysis eFigure 1. Kisspeptin vs Placebo Whole Brain Analysis During Erotic Videos and Facial Attraction Tasks eFigure 2. Erotic Videos: Kisspeptin vs Placebo Whole Brain Activation and Deactivation, Controlled for Visit Order eFigure 3. Effects of Kisspeptin Administration on the Sexual Arousal and Desire Inventory eFigure 4. Effects of Kisspeptin Administration on State Anxiety and Attention eFigure 5. Effects of Kisspeptin on Downstream Circulating Reproductive Hormones [file jamanetwopen-e2236131-s002.pdf]

Supplemental Online Content

Thurston L, Hunjan T, Ertl N, et al. Effects of kisspeptin administration in women with hypoactive sexual desire disorder: a randomized clinical trial. *JAMA Netw Open.* 2022;5(10):e2236131. doi:10.1001/jamanetworkopen.2022.36131

eMethods.

eTable. Clusters With Enhanced Activation or Deactivation by Kisspeptin on Whole Brain Analysis

eFigure 1. Kisspeptin vs Placebo Whole Brain Analysis During Erotic Videos and Facial Attraction Tasks

eFigure 2. Erotic Videos: Kisspeptin vs Placebo Whole Brain Activation and Deactivation, Controlled for Visit Order

eFigure 3. Effects of Kisspeptin Administration on the Sexual Arousal and Desire Inventory

eFigure 4. Effects of Kisspeptin Administration on State Anxiety and Attention

eFigure 5. Effects of Kisspeptin on Downstream Circulating Reproductive Hormones

This supplemental material has been provided by the authors to give readers additional information about their work.

© 2022 Thurston L et al. *JAMA Network Open.*

eMethods

Participants

This study reports on women with HSDD (Group F in the Protocol [Supplement 1]). At screening, the following blood tests were performed to confirm health status: full blood count, renal, liver, bone and thyroid function, LH, FSH, estradiol, progesterone, testosterone, sex hormone binding globulin (SHBG), dehydroepiandrosterone sulphate (DHEAS), and androstenedione. Reproductive hormone levels were consistent with premenopausal status (**Table 1**).

Inclusion criteria included a diagnosis of generalized, acquired HSDD of at least six-month duration in line with DSM-IV-TR criteria[1] and the latest World Health Organization International Classification of Diseases, 11th Edition (ICD-11)[2]. Diagnosis was confirmed with a Female Sexual Function Index (FSFI) score of ≤ 26 (with a desire domain score ≤ 5)[3], as well as a score of ≥ 18 on the Female Sexual Distress Scale – Desire/Arousal/Orgasm (FSDS-DAO) assessment tool[4]. The Patient Health Questionnaire-9 (PHQ-9) and Generalized Anxiety Disorder-7 (GAD-7) questionnaires excluded depression and anxiety, respectively. Mean scores are documented in **Table 1**. Other inclusion criteria were right-handedness, regular menstrual cycles, not taking any form of hormonal contraception, in a stable, monogamous relationship for at least six months prior to their screening appointment, free of current or past psychiatric illness, naïve to psychoactive substances (prescribed or illicit) for a minimum of six months prior to screening, BMI 18.5-30 kg/m^2 and normal or corrected-to-normal vision.

Exclusion criteria were responsive sexual desire (interest in sex that occurs in reaction to sexual stimuli), pregnancy, breastfeeding, history of sexual trauma or abuse, and any contraindication to MRI scanning. No changes to the protocol (including eligibility criteria and sample size) were made after the trial started.

Intervention

Kisspeptin-54 was synthesized and purified by Bachem (Bachem, Switzerland). Vials of kisspeptin-54 were freeze-dried and stored at -20°C and reconstituted in 1ml of 0.9% saline before being added to Gelofusine 4% infusion (B. Braun Medical Ltd, Germany). The kisspeptin dose of 1 nmol/kg/h was selected based on our previous data using intravenous kisspeptin infusions in fMRI studies[5–9]. Placebo (Gelofusine 4% infusion) was administered at a rate equivalent to the kisspeptin infusion.

Assays

Blood samples were collected at the time points as depicted in **Figure 1**A. Plasma kisspeptin immunoreactivity was measured using an established radioimmunoassay, as described previously[10]. Serum LH, FSH, estradiol, progesterone and testosterone were measured using automated chemiluminescent immunoassays (Abbott Diagnostics). Inter-assay coefficients of variation were as follows: kisspeptin 10.2%; LH, 2.7%; FSH, 3.0%; estradiol, 3.0%; progesterone, 2.9%, testosterone 4.6%. Limits of detectability for each assay were as follows: kisspeptin 2 pmol/L; LH 0.05 IU/L; FSH 0.05 IU/L; estradiol 37 pmol/l; progesterone 0.32 nmol/L; testosterone 0.08 nmol/L.

Psychometric questionnaires

Participants were asked to complete a set of questionnaires before (T=-15 minutes) and during (T=70-75 minutes) the kisspeptin or placebo infusion (**Figure 1**A). The Sexual Arousal and Desire Inventory (SADI) was designed to assess subjective sexual arousal and desire based on multiple descriptors categorized into four domains: evaluative (e.g. sexy, excited), negative (aversion, resistant), physiological (e.g. tingling, flushed) and motivational (e.g. lustful, alluring), on a Likert scale of 0 to 5[11]. The STAI Y-1 was designed to assess for any effects of kisspeptin or placebo on state anxiety[12]. The d2 Test of attention was performed at both visits to assess for effects on non-sexual attention as a possible confounder[13].

fMRI procedure

A mirror mounted on the head coil allowed participants to view a screen mounted in the rear of the scanner bore, where visual stimuli were back-projected through a wave guide in the rear wall of the scanner room. Participants also wore headphones to receive instructions and a pulse-oximeter was attached to the participant and connected to a standard data-recording system (AD instruments PowerLab) in the control room. An MRI-compatible response box was used to record participant responses.

Erotic videos task

© 2022 Thurston L et al. *JAMA Network Open.*

Erotic stimuli consisted of 20-second silent erotic videos alternating with neutral non-erotic videos as a control, in a standard, validated block design. During scans, participants were asked to rate their subjective level of arousal on a 20-point scale using a hand-held button box after each video to ensure engagement with the task. The rating period lasted for 5 seconds and was followed by a 10-second blank gray screen, which provided a baseline/rest condition. The erotic videos were the top 10 rated (out of 80 videos) for sexual arousal by an independent focus group comprising 20 healthy heterosexual women. All videos contained one woman and one man engaging in vaginal intercourse (erotic videos) or exercising (control videos). Participants rated the erotic videos as more arousing than the control exercise videos as expected, with no differences observed between kisspeptin and placebo visits.

Facial attraction task

To investigate kisspeptin's effects on brain responses on viewing male versus female high and medium attractiveness faces, participants were presented with 60 images from the validated Chicago Face Database[14]. Thirty faces were from the independently-rated highly attractive category (15 male, 15 female) and 30 faces were from the medium attractive category (15 male, 15 female). Participants were asked to rate the attractiveness of each face on a 5-point Likert scale ranging from "very unattractive" to "very attractive" using a 5-button response box to ensure engagement with the task. We employed an event-related design where each image was presented for 4 seconds, with a jittered intertrial interval of 2-10 seconds to enable effective separation and measurement of the hemodynamic response function (HRF) to each trial. The participants' ratings were concordant with the independent raters, with no differences observed between kisspeptin and placebo visits.

fMRI data acquisition

Imaging data were acquired using a 3T Siemens Trio scanner with a 32-channel, phased-array head coil. Anatomical images were acquired at the beginning of each scan using a T1-weighted magnetization prepared rapid gradient echo (MPRAGE) pulse sequence (1 mm isotropic voxels, repetition time [TR] = 2300 ms, echo time [TE] = 2.98 ms, flip angle = 9°, 160 slices, 256x256 in-plane FOV, bandwidth = 240Hz/pixel, GRAPPA acceleration = 2. For the acquisition of functional images in the videos task, a multiband sequence with acceleration factor 2 (similar to previously validated sequences[25]) was used with the following parameters: 3 mm isotropic voxels, TR = 1250 ms, TE = 30 ms, flip angle = 80°, 44 axial slices, bandwidth = 2232Hz/pixel, GRAPPA acceleration = 2, 192x192 mm FOV.

fMRI data analysis

fMRI data processing was performed using FEAT (fMRI Expert Analysis Tool), part of the Oxford Centre for Functional MRI of the Brain (FMRIB) Software Library (FSL) version 6.0 (www.fmrib.ox.ac.uk/fsl). Registration to high resolution structural images was carried out using the FMRIB Linear Image Registration Tool (FLIRT)[15]. Registration from the high resolution T1 structural image of each participant to standard MNI152 space was then further refined using FMRIB's Nonlinear Image Registration Tool (FNIRT)[16,17]. The following pre-processing steps were applied: motion-correction using motion-correction FLIRT (MCFLIRT)[15], non-brain removal using the Brain Extraction Tool (BET)[18], spatial smoothing (6.0 mm) and high-pass temporal filtering (90-seconds for erotic videos, 100-seconds for facial attraction). All first level models included the extended set of head motion parameters regressors (original parameters, plus derived temporal derivatives and quadratic functions). White matter and cerebrospinal fluid masks were created from each participants' anatomical scans using FMRIB's Automated Segmentation Tool (FAST), and the time series from each functional scan was extracted from these masks for use as a regressor of no interest for each participant in each task to further denoise the data. Time-series statistical analysis was carried out using FMRIB's Improved Linear Model (FILM) with local autocorrelation correction[19]. The analysis approach typically used in task fMRI is a multiple regression method where regressors or explanatory variables are represented in a design matrix, and scaling factors (beta values) are estimated to provide the best fit of each regressor to the data. These beta values can then be contrasted to provide comparisons between regressors (typically representing different task conditions) and statistical values such as t or F values can be derived from the comparisons. The general framework is an application of the General Linear Model (GLM) first described by Friston et al[20]. The regressors of interest were derived from the onset times of the stimulus conditions and were convolved with a gamma function to simulate the HRF. These were used as the main regressors of interest in the GLM with the denoising methods mentioned above as regressors of no interest. The contrasts were defined by each stimulus condition compared to baseline and then also comparing two stimulus conditions of interest. A within-subjects FMRIB's Local Analysis of Mixed Effects (FLAME-1) paired *t*-test was used to investigate differences in whole brain activation on placebo and kisspeptin. A statistical threshold of $Z > 2.3$ and $P = 0.05$ (cluster corrected for multiple comparisons) was used for all fMRI analyses. *A priori* regions of interest (ROIs) were selected for further analysis based on the expression pattern of *KISS1/KISS1R* in the limbic and paralimbic

© 2022 Thurston L et al. *JAMA Network Open.*

system in humans[21,22] and established structures involved in sexual and emotional processing[23,24]. These comprised the amygdala, anterior cingulate, caudate, globus pallidus, hippocampus, insula, nucleus accumbens, posterior cingulate, putamen, and thalamus. ROIs were defined in standard stereotactic space using the Harvard-Oxford cortical and subcortical atlases. The mean of all voxel values within each ROI was extracted from the brain images for each participant per session and correlated with psychometric data.

References

1. American Psychiatric Association. *Diagnostic and Statistical Manual of Mental Disorders: DSM-IV-TR*. 4th, text ed. American Psychiatric Press; 2000.
2. World Health Organization. *International Statistical Classification of Diseases and Related Health Problems*. 11th ed.; 2019.
3. Wiegel M, Meston C, Rosen R. The Female Sexual Function Index (FSFI): Cross-Validation and Development of Clinical Cutoff Scores. *J Sex Marital Ther*. 2005;31:1-20.
4. Derogatis LR, Revicki DA, Rosen RC, Jordan R, Lucas J, Spana C. Psychometric validation of the Female Sexual Distress Scale-Desire/Arousal/Orgasm. *J Patient-Reported Outcomes*. 2021;5(1):100.
5. Comninos AN, Wall MB, Demetriou L, et al. Kisspeptin modulates sexual and emotional brain processing in humans. *J Clin Invest*. 2017;127(2):709-719.
6. Comninos AN, Demetriou L, Wall MB, et al. Modulations of human resting brain connectivity by kisspeptin enhance sexual and emotional functions. *JCI Insight*. 2018;3(20):e121958.
7. Yang L, Demetriou L, Wall MB, et al. Kisspeptin enhances brain responses to olfactory and visual cues of attraction in men. *JCI Insight*. 2020;5(3):e133633.
8. Yang L, Demetriou L, Wall MB, et al. The Effects of Kisspeptin on Brain Response to Food Images and Psychometric Parameters of Appetite in Healthy Men. *J Clin Endocrinol Metab*. 2021;106(4):1837-1848.
9. Comninos AN, Yang L, O'Callaghan J, et al. Kisspeptin Modulates Gamma-Aminobutyric Acid Levels in the Human Brain. *Psychoneuroendocrinology*. 2021;129:105244.
10. Dhillo WS, Chaudhri OB, Patterson M, et al. Kisspeptin-54 Stimulates the Hypothalamic-Pituitary Gonadal Axis in Human Males. *J Clin Endocrinol Metab*. 2005;90(12):6609-6615.
11. Toledano R, Pfaus J. The sexual arousal and desire inventory (SADI): A multidimensional scale to assess subjective sexual arousal and desire. *J Sex Med*. 2006;3(5):853-877.
12. Spielberg CD, Gorsuch RL, Lushene RE. *Manual for the State-Trait Anxiety Inventory*. Consulting Psychologists Press; 1983.
13. Brickenkamp R, Zillmer E. *D2 Test of Attention*. Hogrefe & Huber; 1998.
14. Ma DS, Correll J, Wittenbrink B. The Chicago face database: A free stimulus set of faces and norming data. *Behav Res Methods*. 2015;47(4):1122-1135.
15. Jenkinson M, Bannister P, Brady M, Smith S. Improved optimization for the robust and accurate linear registration and motion correction of brain images. *Neuroimage*. 2002;17(2):825-841.
16. Andersson JLR, Jenkinson M, Smith S. *Non-Linear Optimisation FMRIB Technial Report TR07JA1*.; 2007.
17. Andersson JLR, Jenkinson M, Smith S. *Non-Linear Registration Aka Spatial Normalisation FMRIB Technial Report TR07JA2*.; 2007.
18. Smith SM. Fast robust automated brain extraction. *Hum Brain Mapp*. 2002;17(3):143-155.
19. Woolrich MW, Ripley BD, Brady M, Smith SM. Temporal autocorrelation in univariate linear modeling of FMRI data. *Neuroimage*. 2001;14(6):1370-1386.

© 2022 Thurston L et al. *JAMA Network Open.*

20. Friston KJ, Holmes AP, Poline JB, et al. Analysis of fMRI Time-Series Revisited. *Neuroimage*. 1995;2(1):45-53.

21. Muir AI, Chamberlain L, Elshourbagy NA, et al. AXOR12, a Novel Human G Protein-coupled Receptor, Activated by the Peptide KiSS-1. *J Biol Chem*. 2001;276(31):28969-28975.

22. Kotani M, Detheux M, Vandenbogaerde A, et al. The Metastasis Suppressor Gene KiSS-1 Encodes Kisspeptins, the Natural Ligands of the Orphan G Protein-coupled Receptor GPR54. *J Biol Chem*. 2001;276(37):34631-34636.

23. Stoléru S, Fonteille V, Cornélis C, Joyal C, Moulier V. Functional neuroimaging studies of sexual arousal and orgasm in healthy men and women: A review and meta-analysis. *Neurosci Biobehav Rev*. 2012;36(6):1481-1509.

24. Acevedo BP, Aron A, Fisher HE, Brown LL. Neural correlates of long-term intense romantic love. *Soc Cogn Affect Neurosci*. 2012;7(2):145-159.

25. Demetriou L, Kowalczyk OS, Tyson G, Bello T, Newbould RD, Wall MB. A comprehensive evaluation of increasing temporal resolution with multiband-accelerated protocols and effects on statistical outcome measures in fMRI. *Neuroimage*. 2018;176:404-416.

© 2022 Thurston L et al. *JAMA Network Open.*

eTable. Clusters with Enhanced Activation or Deactivation by Kisspeptin on Whole Brain Analysis

		X	Y	Z	*K*	*Z max*	*P value*
Erotic videos task (erotic > exercise)							
Left inferior/ middle frontal gyrus	Deactivation	-53.9	22.8	19.4	542	3.76	0.0098
Right supramarginal/ postcentral gyrus	Activation	30.3	-21.3	31.1	916	3.73	0.0002
Facial attraction task (male > female)							
Right temporoparietal junction	Deactivation	53.7	-8.83	10.7	496	4.08	0.0160

Data derived from whole brain analysis during the erotic videos and facial attraction task. X, Y and Z are coordinates in a standardized Euclidean space based on the MNI152 brain template and represent the centre of gravity for discrete activation/deactivation clusters observed in the group-level analyses of treatment effects (kisspeptin vs placebo). **X** = sagittal, **Y** = axial, **Z** = coronal, ***K*** = number of voxels per cluster, ***Z* max** = maximum *Z* value of the cluster. *P* values are cluster-wise corrected values (Z=2.3, P<.05) produced by the fMRI analysis software and are the results of within-subjects (paired) t-test comparisons, n=32.

© 2022 Thurston L et al. *JAMA Network Open.*

eFigure 1. Kisspeptin vs Placebo Whole Brain Analysis During Erotic Videos and Facial Attraction Tasks

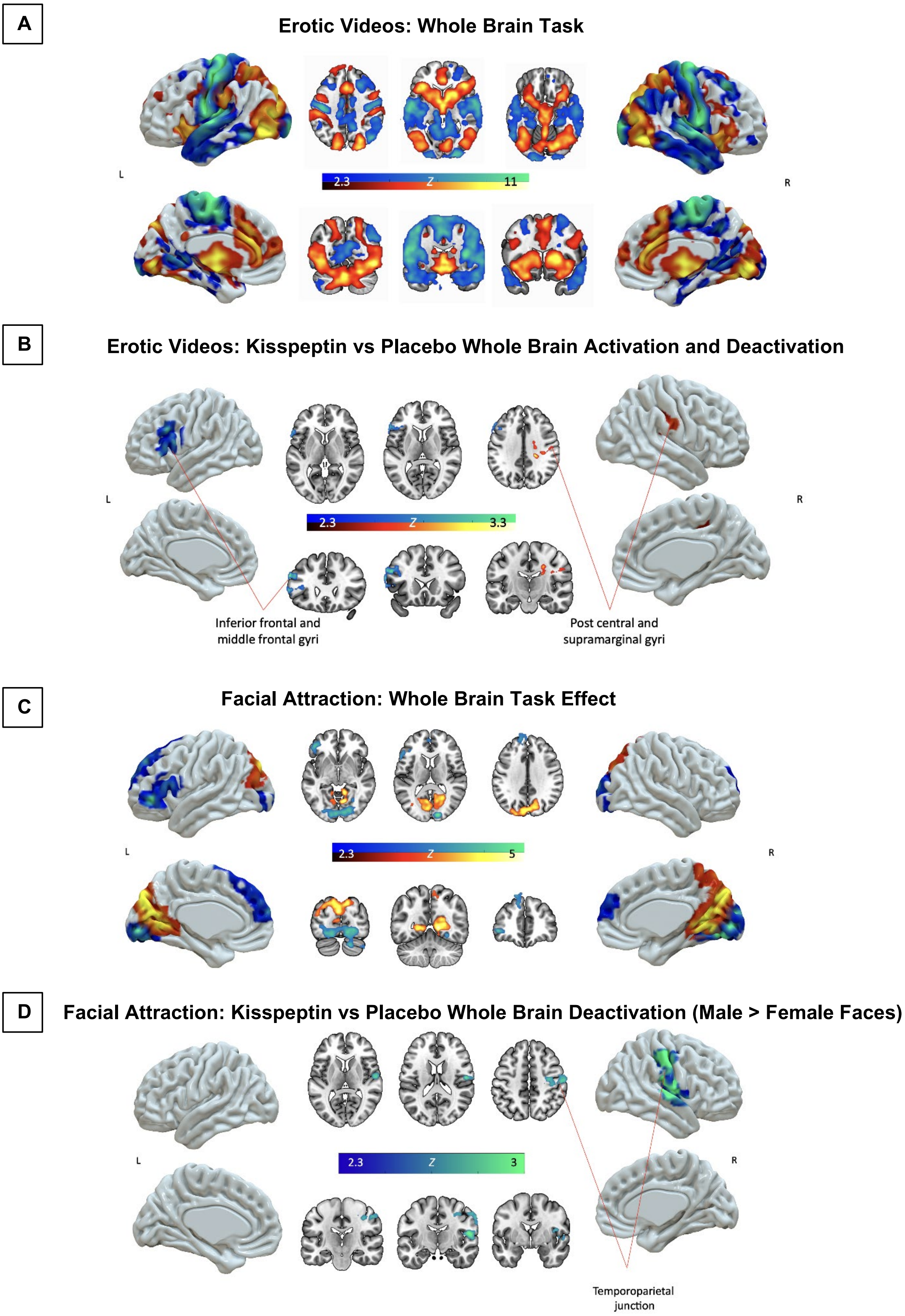

© 2022 Thurston L et al. *JAMA Network Open.*

(**A**) Group average task effects across both kisspeptin and placebo conditions while viewing erotic vs control exercise videos, demonstrating effective task design. Red/yellow regions indicate group activation and blue/green regions indicate group deactivation. *Z*=2.3, *P*<.05. (**B**) Kisspeptin deactivated the inferior frontal and middle frontal gyri and activated the postcentral and supramarginal gyri in response to erotic compared to control exercise videos on whole brain analysis (red/yellow regions indicate group activation and blue/green regions indicate group deactivation). *Z*=2.3, *P*<.05. (**C**) Group average task effects across both kisspeptin and placebo conditions while viewing male vs female faces, demonstrating effective task design. Red/yellow regions indicate group activation and blue/green regions indicate group deactivation. *Z*=2.3, *P*<.05. (**D**) Kisspeptin deactivated the temporoparietal junction in response to male compared to female on whole brain analysis (blue/green regions indicate group deactivation). *Z*=2.3, *P*<.05.

© 2022 Thurston L et al. *JAMA Network Open.*

eFigure 2. Erotic Videos: Kisspeptin vs Placebo Whole Brain Activation and Deactivation, Controlled for Visit Order

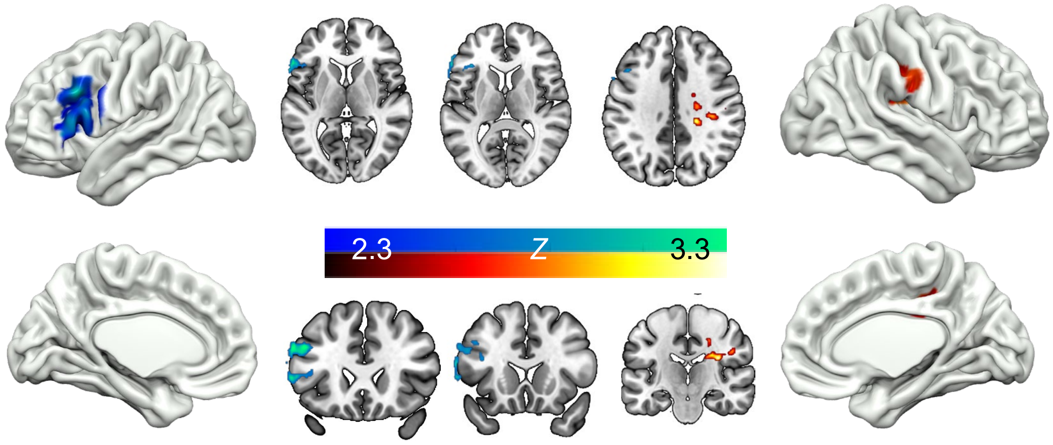

Whole brain analysis of erotic vs control exercise videos using visit order as a demeaned covariate. Data cluster corrected, Z=2.3, *P*<.05, *n*=32.

© 2022 Thurston L et al. *JAMA Network Open.*

eFigure 3. Effects of Kisspeptin Administration on the Sexual Arousal and Desire Inventory

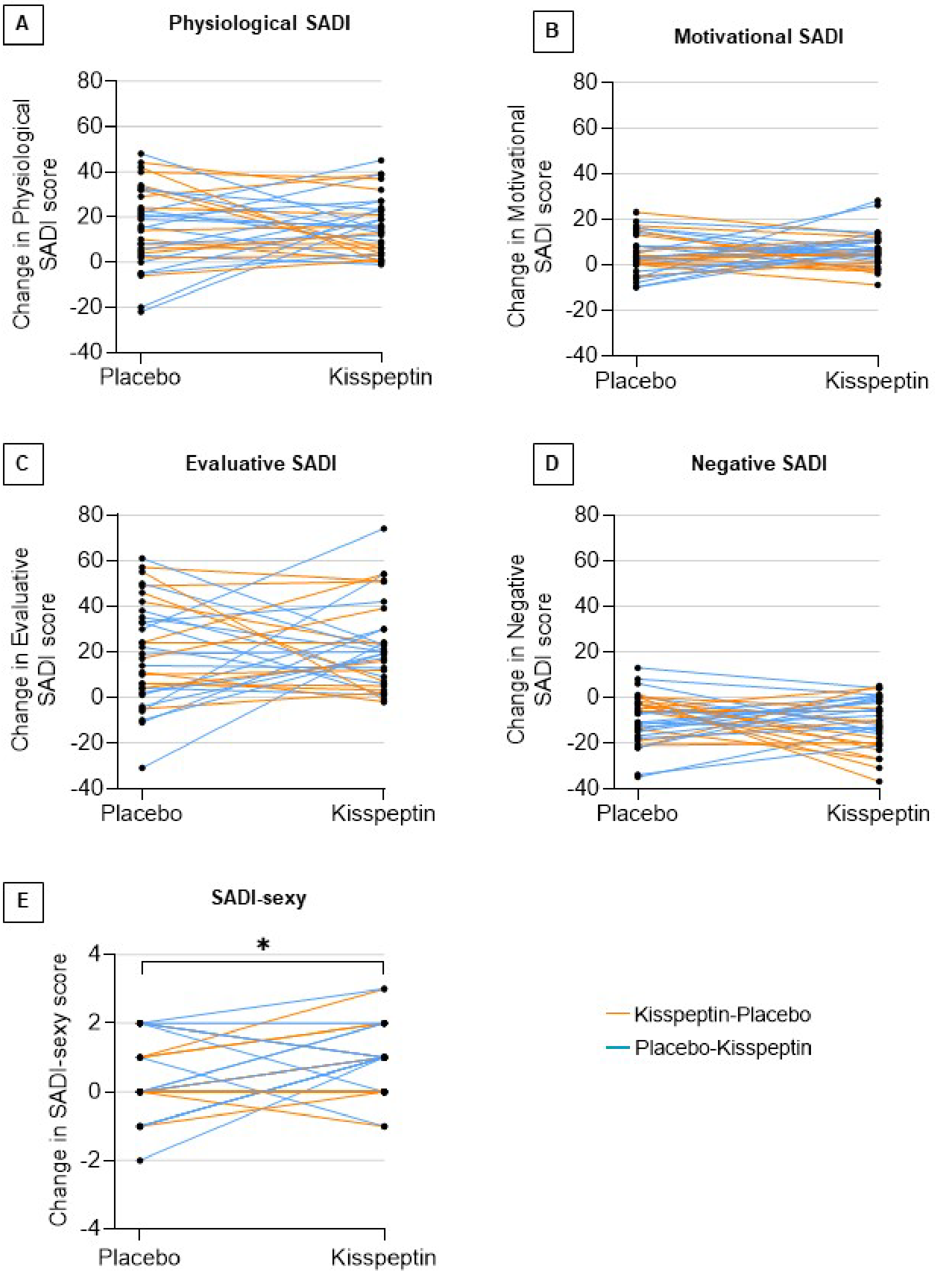

Sexual arousal and desire were assessed during kisspeptin and placebo administration using the SADI. Kisspeptin administration did not alter (**A**) physiological; (**B**) motivational; (**C**) evaluative; or (**D**) negative domains. Post hoc exploratory analysis showed that kisspeptin administration increased (**E**) SADI-sexy score (worded 'how sexy do your feel right now?') by 0.5 (95% CI, 0.05-0.95); P=.04, compared to placebo, Wilcoxon-matched pairs signed rank test. Orange lines depict participants who received kisspeptin at first study visit and placebo at second study visit (n=16). Blue lines depict participants who received placebo at first study visit and kisspeptin at second study visit (n=16). * P<.05, total n=32.

© 2022 Thurston L et al. *JAMA Network Open.*

eFigure 4. Effects of Kisspeptin Administration on State Anxiety and Attention

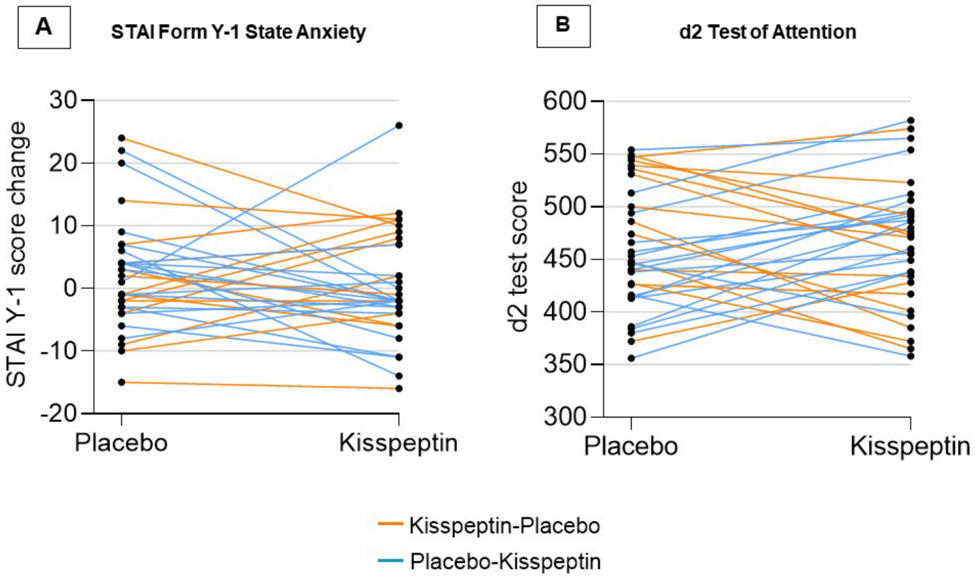

(**A**) State anxiety, assessed using the State-Trait Anxiety Inventory (STAI) Form Y-1, was unaltered following kisspeptin, compared to placebo. (**B**) Non-sexual attention, assessed using the d2 Test, was unaltered during kisspeptin, compared to placebo. Data presented as score change from baseline for each participant. Orange lines depict participants who received kisspeptin at first study visit and placebo at second study visit (*n*=16). Blue lines depict participants who received placebo at first study visit and kisspeptin at second study visit (*n* = 16). Total *n*=32.

© 2022 Thurston L et al. *JAMA Network Open.*

eFigure 5. Effects of Kisspeptin on Downstream Circulating Reproductive Hormones

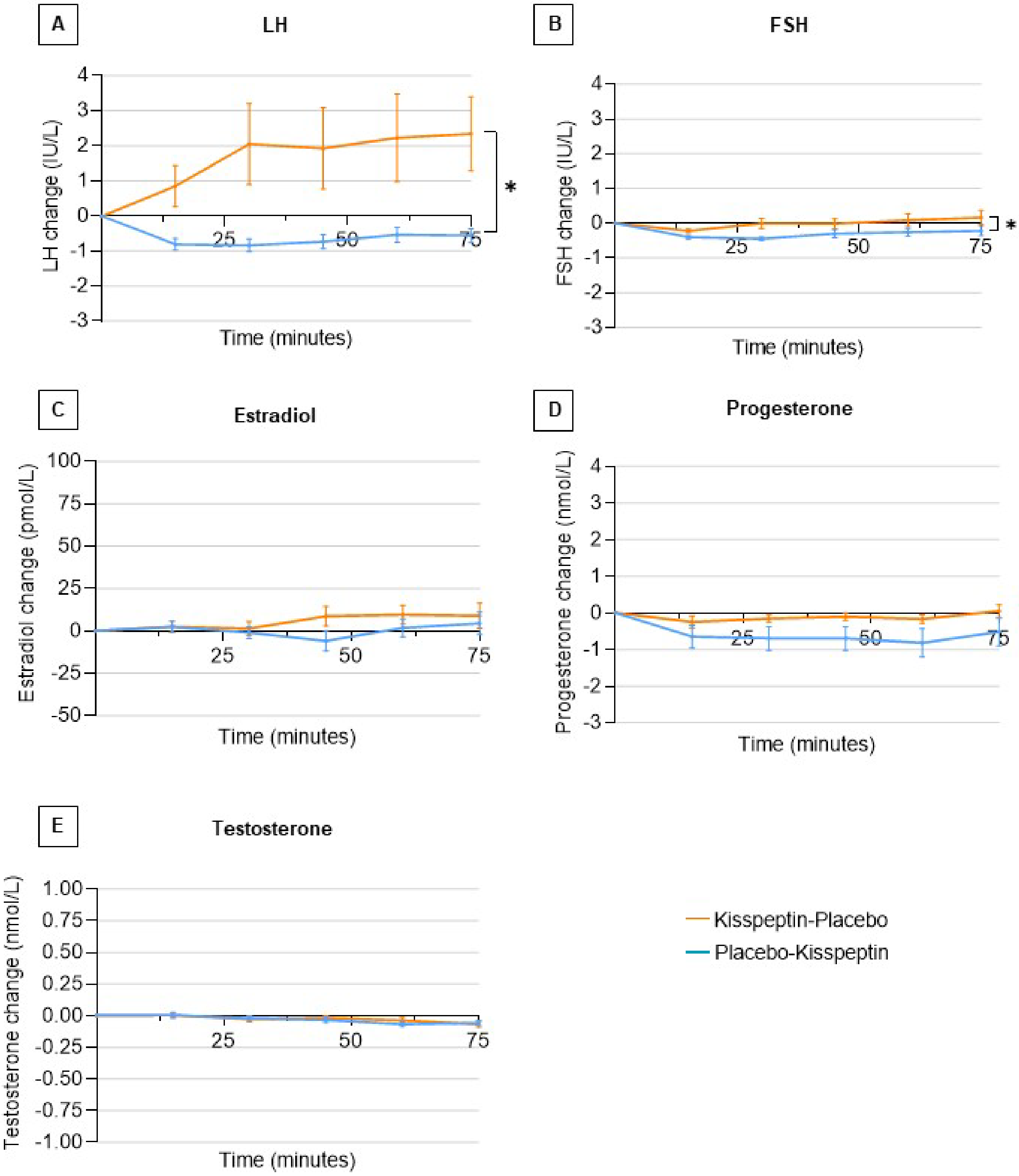

Kisspeptin resulted in an increase in (**A**) LH of 2.14 IU/L (95% CI, 0.41 to 3.90IU/L); F[1,62]=6.08; P=.016) and (**B**) FSH of 0.28 IU/L (95% CI, 0.00 to 0.55IU/L); F[1,62]=104.7; P=.049) across the 75-minute duration of the study, with no effect observed on downstream circulating (**C**) estradiol, (**D**) progesterone, or (**E**) testosterone levels. Data depict mean ± SEM. * P<.05. Data were normally distributed with D'Agostino-Pearson testing, therefore analyzed with two-way ANOVA, n=32.

© 2022 Thurston L et al. *JAMA Network Open.*
