# Supplementary material for: Effects of Kisspeptin Administration in Women With Hypoactive Sexual Desire Disorder: A Randomized Clinical Trial
Source: JAMA Netw Open. 2022 Oct 26;5(10):e2236131. doi: 10.1001/jamanetworkopen.2022.36131 (PMC9606846; doi:10.1001/jamanetworkopen.2022.36131)
Supplement: Supplement 3. — Data Sharing Statement [file jamanetwopen-e2236131-s003.pdf]

Thurston L, Hunjan T, Ertl N, et al. Effects of kisspeptin administration in women with hypoactive sexual desire disorder: a randomized clinical trial. *JAMA Netw Open.* 2022;5(10):e2236131. doi:10.1001/jamanetworkopen.2022.36131

**Data Sharing Statement**

**Data**
**Data available:** No

**Additional Information**
**Explanation for why data not available:** Restrictions apply to the availability of some or all data generated or analyzed during this study to preserve patient confidentiality. The corresponding author will on request detail the restrictions and any conditions under which access to some data may be provided.
